# Cytomorphologic changes in blood erythrocytes, leukocytes, and platelets in dogs progressing through CHOP therapy to treat multicentric lymphoma

**DOI:** 10.1186/s13104-026-07870-y

**Published:** 2026-05-14

**Authors:** Joseph DiBenedetto, Laura Van Vertloo, Margaret Musser, Chad Johannes, Ariel Nenninger, Shannon Jones Hostetter, Austin Keith Viall

**Affiliations:** 1 Red Bank Veterinary Hospital Mount Laurel, 2061 Briggs Road, Mount Laurel Township, NJ USA; 2 Iowa State University, 1800 Christensen Drive, Ames, IA USA; 3 Colorado State University, 300 W. Drake Road, Fort Collins, CO USA; 4 University of Georgia, 501 DW Brooks Drive , Athens, GA USA; 5 University of California Davis, One Shields Avenue, Davis, CA USA

**Keywords:** Chemotherapy, Cyclophosphamide, Doxorubicin, Lymphoma, Myelodysplasia, Vincristine

## Abstract

**Objective:**

Chemotherapeutic agents can induce morphologic changes in erythrocytes, leukocytes, and platelets in peripheral circulation. However, the cytomorphologic alterations associated with chemotherapy administration in circulating hemic cells are minimally characterized in dogs. Study objectives were to (1) assess differences in erythrocyte, leukocyte, and platelet morphologies and CBC metrics between healthy matched control dogs and dogs with multicentric lymphoma and (2) evaluate alterations in hemic cell morphology and CBC metrics in dogs with multicentric lymphoma being treated with multiagent CHOP chemotherapy regimen.

**Results:**

Dogs with multicentric lymphoma (*n* = 20) had no differences in frequency and magnitude of hemic cell cytomorphology compared to control dogs (*n* = 20). However, lymphoma dogs had higher leukocyte, neutrophil, and monocyte counts. As dogs with lymphoma progressed through CHOP therapy, no differences in leukocyte or platelet cytomorphology were observed. The frequency and magnitude of erythrocyte polychromasia and were different between multiple chemotherapy timepoints. No morphologies consistent with dysplastic change were observed for any lineages. Differences in several erythrocyte, leukocyte, and platelet CBC indices were also observed. Atypical cytomorphologic abnormalities in circulating erythrocytes, leukocytes, and platelets, with the exception of leukemic cells, may be infrequent findings in dogs with multicentric lymphoma being treated with CHOP chemotherapy.

**Supplementary Information:**

The online version contains supplementary material available at 10.1186/s13104-026-07870-y.

## Introduction

Lymphoma is the most common hemic-origin neoplasm in dogs, representing 13–24% of all canine cancer cases with a lifetime incidence of 64–114 cases per 100,000 dogs [1–3]. Chemotherapy protocols that consist of cyclophosphamide, doxorubicin, vincristine, and prednisone (CHOP) are commonly used chemotherapy regimens to treat multicentric, high grade canine lymphoma regardless of tumor immunophenotype, with T-cell lymphomas also commonly treated with alkylating agent-rich regimens such as LOPP and MOPP [4, 5]. Madison-Wisconsin CHOP protocols, which alternate chemotherapeutic agent administration across 12, 15, 19 or 25 week-long protocols, are most frequently utilized and associated with median survival time of 302–321 days for multicentric large B-cell lymphoma and 159–235 days for multicentric large T-cell lymphoma [6–8]. Performing serial complete blood counts (CBC) is essential for dogs being treated with CHOP chemotherapy to monitor for chemotherapy-induced cytopenia, lymphoma progression to leukemic phase, adverse drug reactions, and systemic health. Some chemotherapeutics, like vinca alkaloids, are documented to induce morphological alterations in erythrocyte, leukocyte, or platelet lineages in circulation and bone marrow [9, 10]. Agent-induced cytomorphological changes can closely overlap with both dysplastic changes secondary to cancer-associated dysmyelopoiesis or a true neoplastic phenotype [11, 12]. The frequency and spectrum of potential hemic cell cytomorphological changes caused by CHOP chemotherapeutic agents in dogs are minimally reported. Characterizing the leukocyte, platelet, and erythrocyte morphological alterations in dogs with multicentric CHOP may help differentiate expected cytomorphological changes from abnormalities suggesting unexpected adverse chemotherapy side effects, disease progression, or tumor-associated sequala.

We conducted a preliminary investigation to describe cytomorphologic changes in peripheral circulating erythrocytes, leukocytes, and platelets in dogs with multicentric lymphoma treated with a CHOP protocol. We hypothesized that dogs with lymphoma would demonstrate cytomorphologic alterations consistent with dyserythropoiesis and dysgranulopoiesis, potentially attributable to chemotherapy-induced changes, as they progress through the chemotherapy regimen.

## Methods

### Study design

Retrospective study performed at Lloyd Veterinary Medical Center, College of Veterinary Medicine, Iowa State University with cases and controls collected between April 1, 2005 - December 31, 2022.

### Animals and case selection

Medical records of dogs diagnosed and treated for multicentric lymphoma from January 24, 2005, to July 16, 2016, were retrospectively reviewed. Case inclusion criteria were (a) diagnosis of lymphoma by cytology, histopathology with and without immunohistochemistry, lymphocyte clonality testing, and/or flow cytometric immunophenotyping, (b) diagnostic findings consistent with multicentric tumor distribution, (c) CBC performed at hospital-associated clinical pathology laboratory prior to initiating chemotherapy and throughout duration of CHOP therapy, (d) treatment naïve at start of CHOP, and (e) completion of CHOP protocol (Table 1). Cases in which chemotherapeutic agent substitutions within drug class were performed were considered acceptable for inclusion. Dogs were excluded if they did not demonstrate and sustain clinical remission after initiating CHOP therapy.

**Table 1:** 25-week CHOP protocol regimen and study timepoints

25 Week CHOP Protocol												
Treatment Week	1	2	3	4	5	6	7	8	9	10	11	12
Study Timepoint	V1	C1	V2	D1		V3	C2	V4	D2		V5	
Chemotherapy Agent (Dose)												
Vincristine (0.5 mg/m^2^—0.7 mg/m^2^ IV)	X		X			X		X			X	
Cyclophosphamide (200—250 mg/m^2^ PO)		X					X					
Doxorubicin (30 mg/m^2^ IV)				X					X			
Prednisone (2.0—0.5 mg/kg PO q24h)	2.0 mg /kg	1.5 mg /kg	1.0 mg /kg	0.5 mg /kg								

25 Week CHOP Protocol													
Treatment Week	13	14	15	16	17	18	19	20	21	22	23	24	25
Study Timepoint	C3		V6		D3		V7		C4		V8		D4
Chemotherapy Agent (Dose)													
Vincristine (0.5 mg/m^2^—0.7 mg/m^2^ IV)			X				X				X		
Cyclophosphamide (200—250 mg/m^2^ PO)	X								X				
Doxorubicin (30 mg/m^2^ IV)					X								X
Prednisone (2.0—0.5 mg/kg PO q24h)													

Vincristine, cyclophosphamide, and doxorubicin administered on weeks indicated by X. Prednisone administered during Weeks 1–4 at indicated doses

To find dogs to serve as matched, non-lymphoma controls, the medical record database was searched from January 1, 2011, to December 31, 2022, to identify dogs with the following criteria: (a) breed- and age-matched to respective lymphoma dog, (b) were considered to be healthy based upon review of medical record, and (c) had at least one CBC performed.

### Data collection

The following data was collected from medical records for lymphoma patients: signalment, physical examination, current medications, CBC, and blood cell cytomorphology data for all timepoints through CHOP protocol. Data collected for control dogs included: signalment, physical examination, current medications, and CBC with cytomorphology data on day of presentation.

Presence and magnitude of the following cytomorphologic changes were documented. Erythrocytes: polychromasia, echinocytosis, codocytosis, elliptocytosis, keratocytosis, acanthocytosis, macrocytosis, spherocytosis, Howell-Jowell bodies, schistocytosis, basophilic stippling, hypochromasia, eccentrocytosis, microcytosis, dacrocytosis, nucleated RBCs (nRBC), ghost cell, stomatocytosis, Heinz bodies, rouleaux, agglutination, siderocytosis, and atypical nuclear formations in nRBCs. Leukocytes: overall neutrophil toxic change, toxic vacuoles, toxic basophilia, toxic granulation, reactive lymphocytes, atypical lymphocytes, granular lymphocytes, granulocyte nuclear atypia, and large unclassified cells. Platelets: macroplatelets, megakaryocytes, and atypical platelet morphology. Credentialed medical technologists or board-certified veterinary clinical pathologists performed standardized blood smear review. The magnitude of morphologic abnormalities was scored according to a standardized rubric (Supplemental Table 1) and converted to a numerical score for statistical comparisons: none = 0, mild = 1, moderate = 2, severe = 3. The following CBC metrics were collected: white blood cell count (WBCC), red blood cell count (RBCC), hemoglobin concentration (Hgb), hematocrit (HCT), mean corpuscular volume (MCV), mean corpuscular hemoglobin (MCH), mean corpuscular hemoglobin concentration (MCHC), red cell distribution width (RDW), reticulocyte count, platelet count (PLTC), mean platelet volume (MPV), specific leukocyte counts, absolute large unclassified cells, nucleated RBCs, and leukocyte percentages. For dogs with lymphoma, all data was categorized relative to the week of CHOP protocol (Table 1).

### Statistical analyses

Continuous data parameters were evaluated by D’Agostino Pearson analysis for normality. To assess differences in cytomorphologies and CBC metrics between lymphoma dogs at diagnosis relative to matched controls, Fischer’s Exact analysis was used to evaluate frequency and paired T-test or matched Wilcoxon rank analysis. For lymphoma dogs only, differences in cytomorphology magnitude and CBC metrics were assessed across all CHOP timepoints, including presentation, by a mixed-effects model with Tukey correction for multiple comparisons. Differences in morphology frequencies were evaluated by fraction of total analysis with a 95% confidence interval overlap assessment, followed by Fisher’s Exact analysis with Bonferonni correction. Significance was defined as *P* < 0.05. Statistical analyses were performed using commercial software (Prism 10. GraphPad Software LLC). Data is presented as mean (standard deviation) or median (range) or and percentage of cases with abnormality for frequency.

## Results

Twenty dogs with lymphoma met the inclusion criteria. Nine were castrated males, two were intact males, and nine were spayed females. Lymphoma cases had a median age of 7.1 years (3.5–12.0); 9/20 had B-cell lymphoma, 1/20 had T-cell lymphoma, and 10/20 did not have immunophenotyping performed. Breeds represented included Pembroke Welsh Corgi (*n* = 5), Mixed Breed Dog (*n* = 3), Bernese Mountain Dog (*n* = 2), Golden Retriever (*n* = 2), and one each of the following: Beagle, Border Collie, Doberman, Labrador Retriever, Samoyed, Siberian Huskey, Staffordshire Terrier, and Rottweiler. Alterations to the CHOP protocol occurred for 17/20 dogs, with changes either being delays in chemotherapy administration or agent substitution. Twenty dogs were identified as matched controls. The control population included nine castrated males, one intact male, nine spayed females, and one intact female. The control population had identical breed demographical data as lymphoma dogs, with median age 7.25 years (3.0–12.0).

No differences in the frequency or magnitude of cytomorphologic abnormalities for erythrocytes, leukocytes, or platelets were found between lymphoma dogs at initial presentation relative to matched controls (Supplemental Table 2). At diagnosis, dogs with lymphoma had a higher white blood cell count, higher neutrophil count, higher monocyte count, and a higher %monocyte than healthy dogs (Supplemental Fig. 1; Supplemental Table 3).

**Fig. 1:**
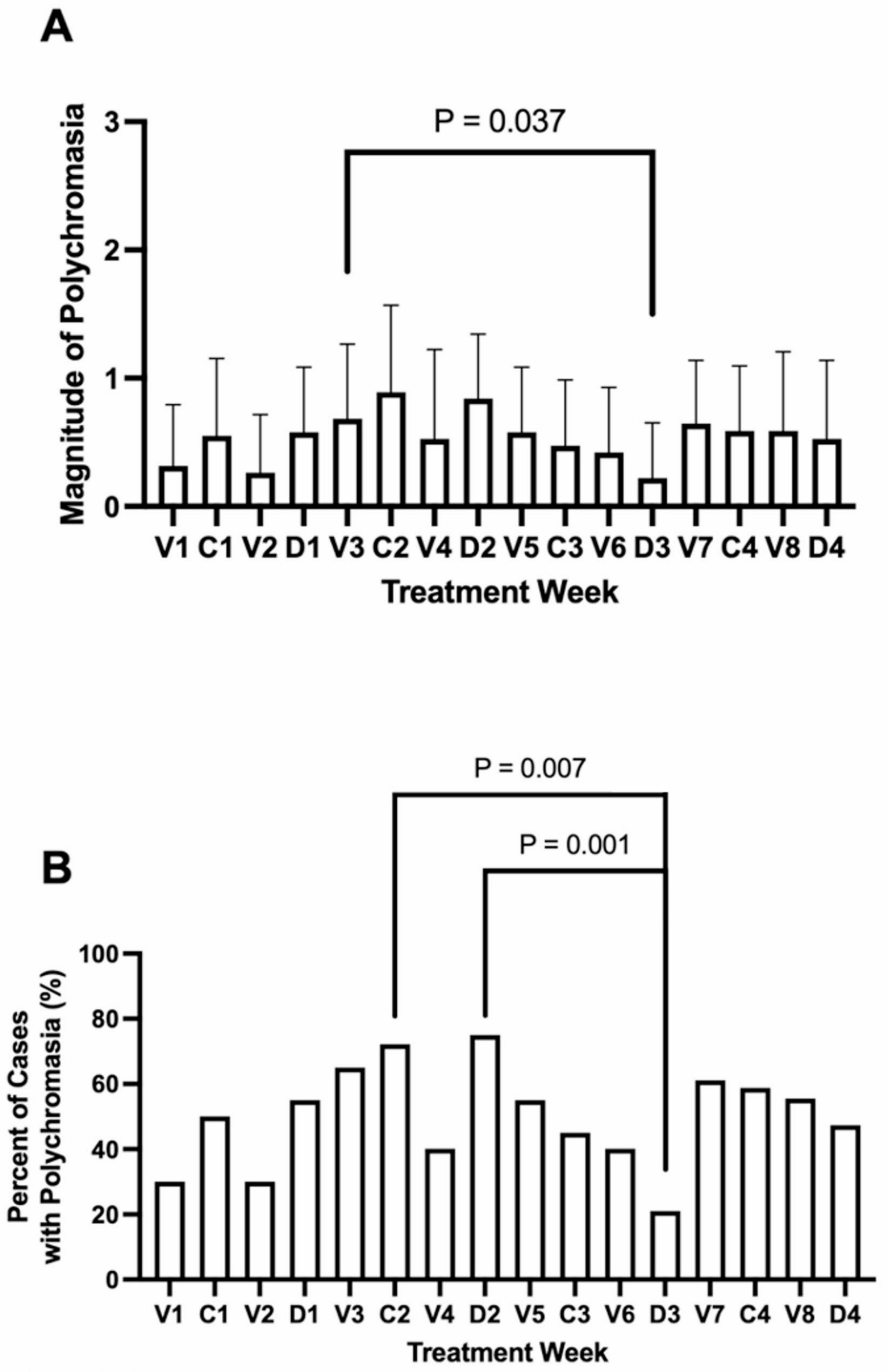
Differences in erythrocyte morphology in dogs with multicentric lymphoma progressing through CHOP therapy. The magnitude (**A**) and frequency (**B**) of polychromasia were lower at D3 compared to V3, and at C2 and D2, respectively, in the CHOP regimen.

As dogs with lymphoma progressed through CHOP therapy, there were differences in the frequency and magnitude of erythrocyte polychromasia. Magnitude of polychromasia was lower at V3 compared to D3 (*P* = 0.037) and polychromasia was documented less frequently D3 relative to C2 and D2 (*P* = 0.007 and *P* = 0.001, respectively) (Fig. 1 and Supplemental Tables 4 and 5). Abnormal nRBC morphology was not observed for any cases. No differences in leukocyte or platelet cytomorphologies, frequencies, or magnitudes were observed across any timepoints, and no atypical features were observed in any platelets, granulocytes, or monocytes (Supplemental Tables 3 and 4). Large unclassified cells, likely representing circulating neoplastic lymphocytes, were identified in 2 dogs at different timepoints.

Erythron parameters which changed throughout CHOP therapy included RBCC, Hgb, MCV, MCH, and RDW (Supplemental Fig. 2 and Supplemental Table 6). Concerning leukogram metrics, there were differences in WBCC, neutrophil count, %neutrophil, monocyte count, %monocyte, eosinophil count, %eosinophil, and %lymphocyte% observed throughout CHOP (Supplemental Fig. 3; Supplemental Table 6). Numerous differences were observed in platelet count throughout CHOP as well (Supplemental Fig. 4; Supplemental Table 6).

## Discussion and conclusions

Our investigation sought to determine the frequency and severity of cytologic atypia in circulating erythrocytes, leukocytes, and platelets in dogs with multicentric lymphoma being treated with a CHOP protocol. Minimal changes in erythrocyte morphology and no difference in leukocytes and platelet morphology were found as dogs progressed through chemotherapy. There were no differences in hemic cell morphologies between lymphoma dogs and control dogs. Additionally, no morphologic abnormalities that would be considered atypical or dysplastic and potentially attributable to chemotherapy administration were observed in any hemic cell lines. Our findings suggest that morphological atypia in erythrocytes, leukocytes, and platelets – except for circulating neoplastic cells – are not routinely observed in dogs with lymphoma receiving CHOP therapy.

We documented several differences in RBC, WBC, and platelet metrics as dogs with multicentric lymphoma progress through CHOP chemotherapy. Changes found among RBC indices are supportive of initial and potentially sustained regenerative erythropoiesis throughout therapy. WBC alterations suggest that episodic granulocyte suppression and regeneration also occur throughout the chemotherapy regimen. An initial increase in platelet density in the early phase of CHOP therapy but gradual decrease in platelet density later in the regimen may occur in CHOP therapy. Relative to control dogs, at presentation dogs with lymphoma have leukogram changes supportive of a more inflammatory or corticosteroid response associated state.

Our original impetus for this study came from a dog with lymphoma being treated with chemotherapy that had neutrophils and rubricytes with significant nuclear atypia. Agents used in CHOP protocol have documented adverse hematologic effects in dogs, including induction of multilineage cytopenias, dysmyelopoiesis, and hemic cell morphologic atypia [9, 10, 12–15]. Doxorubicin can induce echinocytosis along with a post-administration regenerative anemia, which can be associated with polychromasia [14]. Dogs treated with vincristine can develop dyserythropoiesis associated with atypical nuclear configurations in erythroid precursors in bone marrow and circulation [9, 10]. Erythroid lineage may be particularly susceptible to the mitotic S-phase related effects of vincristine as 60% of erythroid cells in the marrow maintain mitotic potential [9]. Dysmegakaryopoiesis has previously been reported in a dog with lymphoma on CHOP chemotherapy [15]. Bone marrow level dysgranulopoiesis is also reported to occur secondary to chemotherapy in dogs with neoplasia; neutrophil nuclear hypolobulation and pseudo-Pelger Huët anomaly is documented [11, 12, 16]. The presence of erythrocyte, leukocyte, or platelet atypia in dogs with cancer treated with chemotherapy could reflect individual drug-induced changes, idiosyncratic drug response, neoplasia-induced dysmyelopoiesis, or circulating neoplastic cells.

Frequency and magnitude of morphologic atypia in circulating hemic cells in dogs treated for lymphoma is minimally characterized. Cora et al. reported that clinically relevant morphological abnormalities were observed in 20% of blood smears from a heterogeneous population of dogs treated for various cancers [17]. Our results similarly support atypical morphologies in non-neoplastic circulating erythrocytes, leukocytes, and platelets are uncommon in dogs with lymphoma. The only significant changes in cytomorphology were limited to erythrocytes and were supportive of periods of erythroid series regeneration, aligning with the erythrogram changes [18]. Anemia occurs in 32% of dogs with lymphoma, with multiple pathophysiologic mechanisms contributing including a chronic inflammatory state, iron deficiency, and chemotherapy-induced erythrosuppression [11, 19, 20]. While these changes are clinically relevant for canine patients and may indicate erythroid series rebound due to decreased tumor burden or post-chemotherapy administration, such features are not considered dysplastic or atypical erythrocyte morphologies.

Apart from circulating neoplastic cells, atypical morphology of erythrocytes, leukocytes, and platelets appear to be infrequent findings in dogs with multicentric lymphoma treated with CHOP therapy. Presence of such atypical features in dogs with lymphoma could represent idiosyncratic or adverse responses to chemotherapeutics or neoplasia sequela.

## Limitations

Our investigation has limitations inherent to the retrospective nature of study design. While all dogs with multicentric lymphoma completed CHOP therapy regimen, there were differences in treatment plan as CHOP protocol alterations were made on case-by-case basis due to differences in response to chemotherapy, chemotherapeutic drug tolerance, and owner compliance. Certain chemotherapeutic drugs were substituted for drugs of a similar mechanistic class due to patient tolerance. Such treatment variability could have affected our ability to identify statistically different changes in cellular morphology as dogs progressed through CHOP, but would not likely have affected identification of atypical or dysplastic changes on blood smear review. While a standardized rubric was utilized by the clinical pathology laboratory to identify and quantitate cytomorphologic abnormalities and all laboratory members were highly trained hematologic diagnosticians, inter-personnel differences in expertise could have influenced identification and documentation of cytomorphologic abnormalities. In a prospective follow-up investigation, review of blood smears by two boarded veterinary clinical pathologists would help address such diagnostic variability along with achieving a consensus assessment of morphologic changes. Our patient population was also comprised of dogs diagnosed with both multicentric B- and T-cell lymphoma. While CHOP is an acceptable therapy for both types of lymphoma, B- and T-cell lymphomas have distinct and highly heterogeneous biological behaviors and could influence results. Given the variable biological nature of canine lymphoma, our comparatively low number of evaluated cases, and the absence of cases with significant hematologic dyscrasias due to their neoplasia at the onset of treatment, our pilot study findings may not be fully representative of the spectrum and frequency of chemotherapy-induced cytomorphologic changes possible in dogs with large cell lymphoma being treated with CHOP.

## Electronic Supplementary Material

Below is the link to the electronic supplementary material.

Supplementary Material 1.

## Data Availability

Authors are amenable to the distribution of raw data with reasonable request.

## Abbreviations

CBC: Complete blood count
CHOP: Cyclophosphamide, doxorubicin, vincristine, and prednisone
HCT: Hematocrit
Hgb: Hemoglobin
MCH: Mean corpuscular volume
MCHC: Mean corpuscular hemoglobin concentration
MCV: Mean corpuscular volume
MPV: Mean platelet volume
nRBC: Nucleated red blood cell
RBC: Red blood cell
RBCC: Red blood cell count
PLTC: Platelet count
RDW: Red cell distribution width
WBCC: White blood cell count
