# Supplementary material for: Cytomorphologic changes in blood erythrocytes, leukocytes, and platelets in dogs progressing through CHOP therapy to treat multicentric lymphoma
Source: BMC Res Notes. 2026 May 14;19:282. doi: 10.1186/s13104-026-07870-y (PMC13343946; doi:10.1186/s13104-026-07870-y)

**Supplemental Figure 1. Differences in hematologic parameters between dogs with multicentric lymphoma at diagnosis and control dogs.** Dogs with multicentric lymphoma had higher leukocyte count, neutrophil count, monocyte count, and percentage of circulating monocytes compared to control dogs.


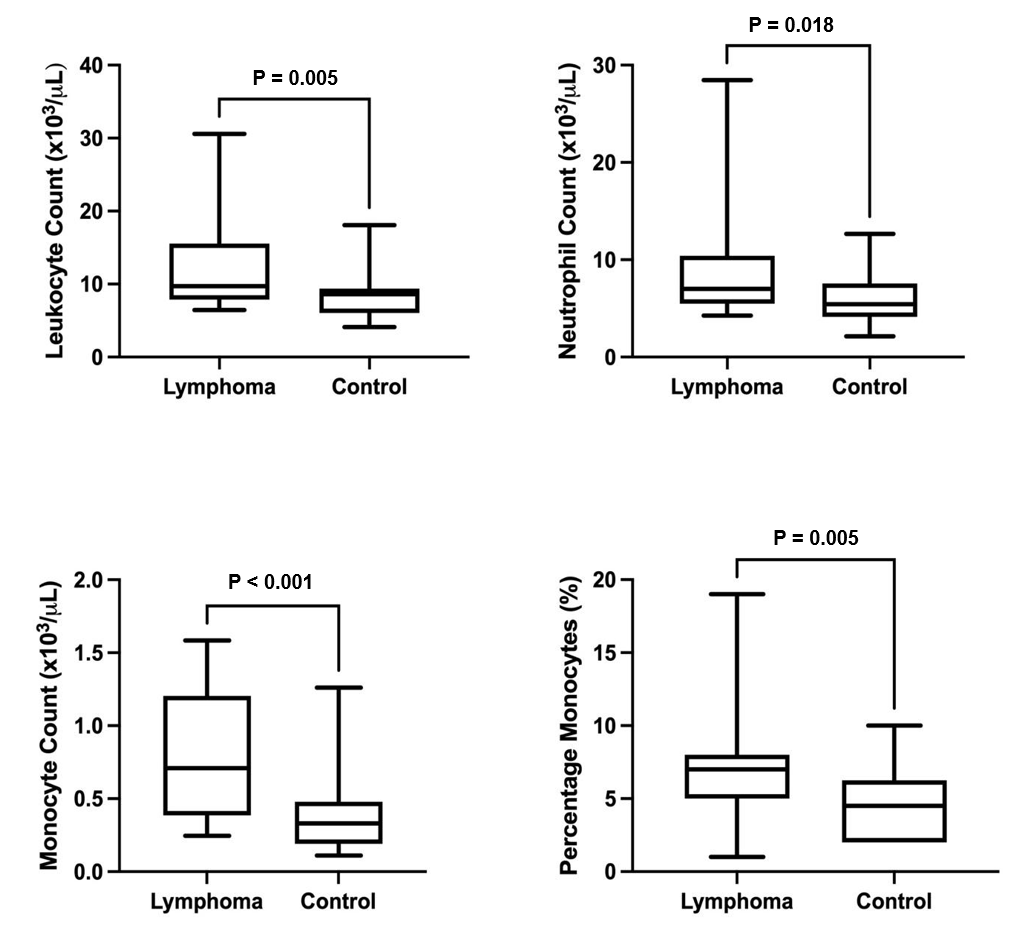

Supplement: Supplementary file 1 — Supplementary Material 1. [file 13104_2026_7870_MOESM1_ESM.zip › Supplementary/Supplemental Figure 1.docx]
