# Supplementary material for: Cytomorphologic changes in blood erythrocytes, leukocytes, and platelets in dogs progressing through CHOP therapy to treat multicentric lymphoma
Source: BMC Res Notes. 2026 May 14;19:282. doi: 10.1186/s13104-026-07870-y (PMC13343946; doi:10.1186/s13104-026-07870-y)
Supplement: Supplementary file 1 — Supplementary Material 1. [file 13104_2026_7870_MOESM1_ESM.zip › Supplementary/Supplemental Figure 2.docx]

**Supplemental Figure 2. Changes in erythron parameters as dogs with multicentric lymphoma progress through CHOP therapy.** For timepoint V1, there was differences in RBCC, hemoglobin, MCH, and RDW relative to several other CHOP timepoints.

**
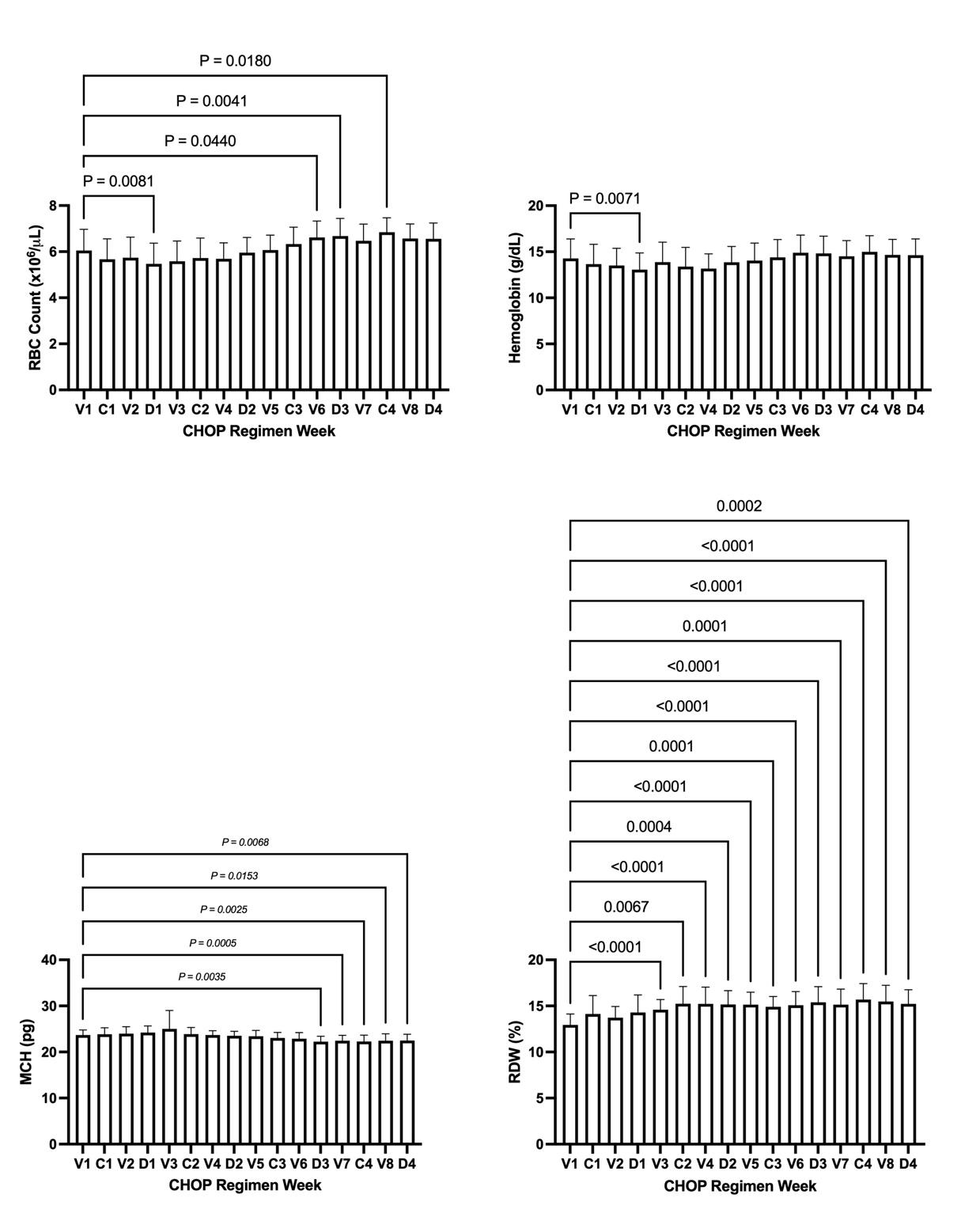
**
