# Supplementary material for: Cytomorphologic changes in blood erythrocytes, leukocytes, and platelets in dogs progressing through CHOP therapy to treat multicentric lymphoma
Source: BMC Res Notes. 2026 May 14;19:282. doi: 10.1186/s13104-026-07870-y (PMC13343946; doi:10.1186/s13104-026-07870-y)

**Supplemental Figure 3. Differences in leukocyte metrics for dogs with multicentric lymphoma progressing through CHOP therapy.** Differences in leukocyte count, neutrophil count, %neutrophils, monocyte count, %monocytes, eosinophil count, %eosinophils, %lymphocytes were observed between various timepoints in CHOP therapy.


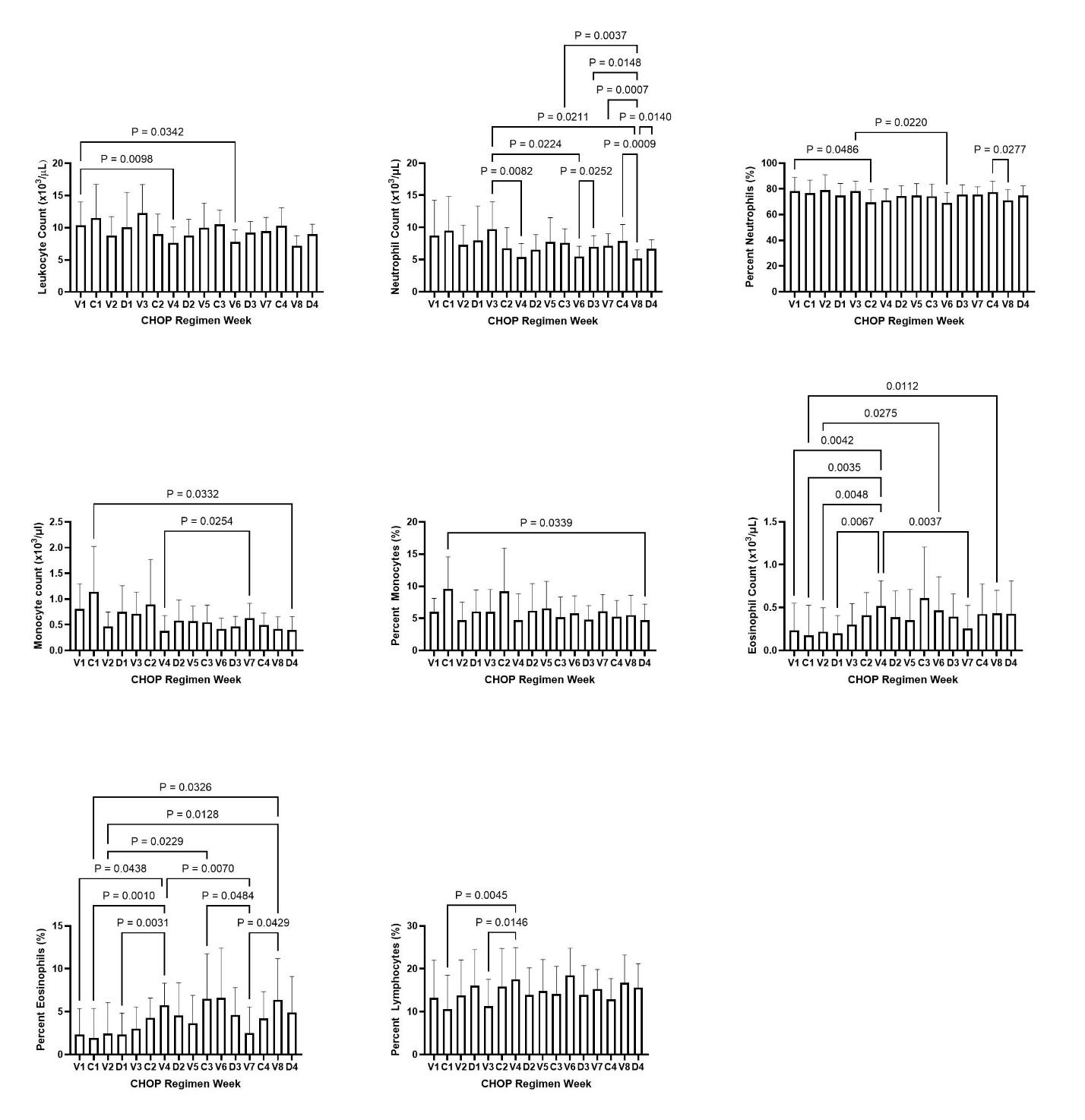

Supplement: Supplementary file 1 — Supplementary Material 1. [file 13104_2026_7870_MOESM1_ESM.zip › Supplementary/Supplemental Figure 3.docx]
