# Supplementary material for: Cytomorphologic changes in blood erythrocytes, leukocytes, and platelets in dogs progressing through CHOP therapy to treat multicentric lymphoma
Source: BMC Res Notes. 2026 May 14;19:282. doi: 10.1186/s13104-026-07870-y (PMC13343946; doi:10.1186/s13104-026-07870-y)

**Supplemental Figure 4. Changes in platelet count for dogs with multicentric lymphoma progressing through CHOP therapy.** Platelet counts at V2 and C2 were increased relative to several timepoint towards the beginning and end of the CHOP protocol.


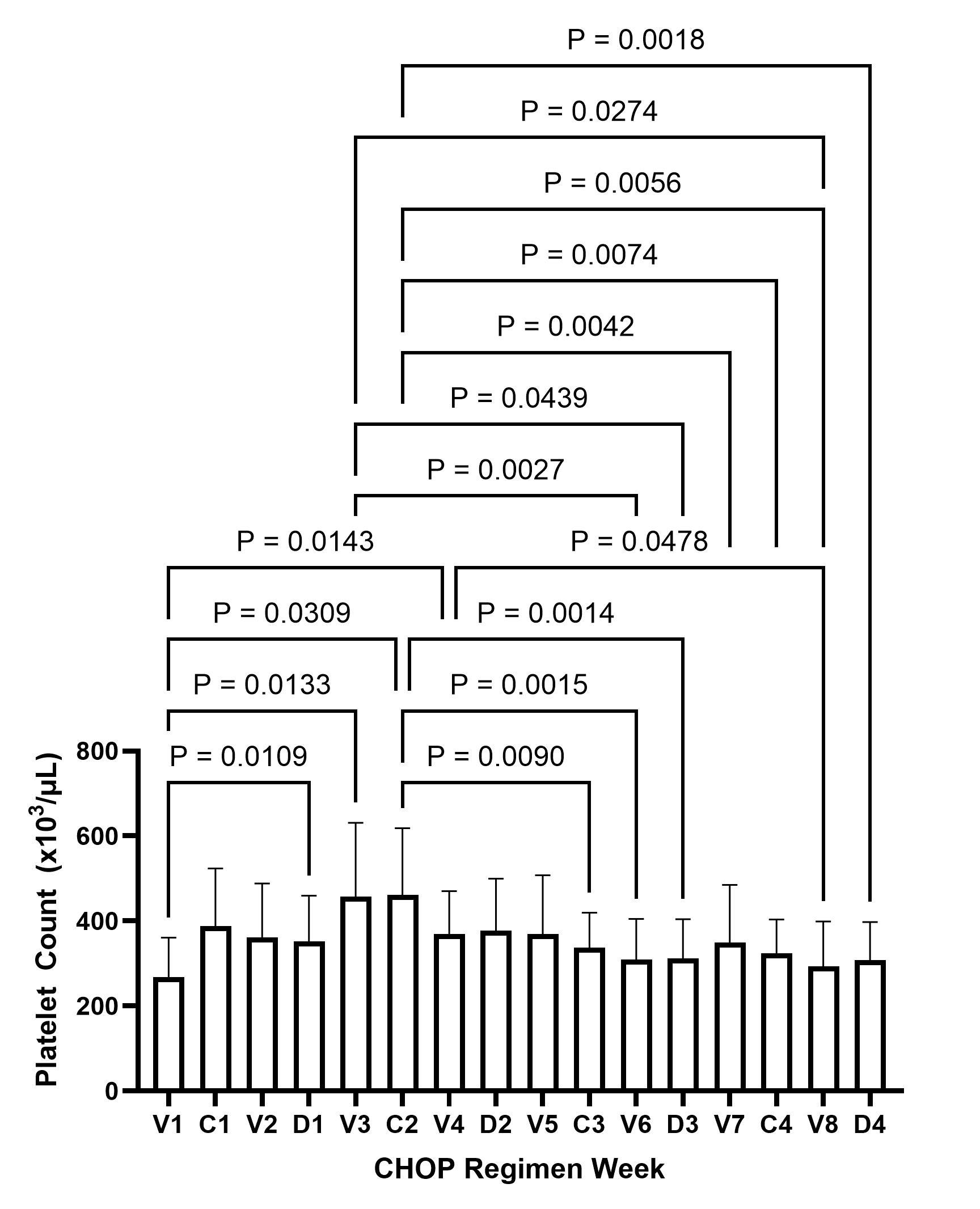

Supplement: Supplementary file 1 — Supplementary Material 1. [file 13104_2026_7870_MOESM1_ESM.zip › Supplementary/Supplemental Figure 4.docx]
