# Supplementary material for: Cytomorphologic changes in blood erythrocytes, leukocytes, and platelets in dogs progressing through CHOP therapy to treat multicentric lymphoma
Source: BMC Res Notes. 2026 May 14;19:282. doi: 10.1186/s13104-026-07870-y (PMC13343946; doi:10.1186/s13104-026-07870-y)
Supplement: Supplementary file 1 — Supplementary Material 1. [file 13104_2026_7870_MOESM1_ESM.zip › Supplementary/Supplemental Table 1 Updated.docx]

**Supplemental Table 1. Erythrocyte, leukocyte, and platelet morphology quantification rubric and scoring scheme.**

| **Erythrocyte Morphology** | **Absent or normal density (Score = 0)** | **Mildly increased (Score = 1)** | **Moderately increased (Score = 2)** | **Markedly increased (Score = 3)** |
| --- | --- | --- | --- | --- |
|  | *Quantitated as number of erythrocytes with specific morphology in ten 1000x magnified fields.* | | | |
| Polychromasia | <2 | 2-7 | 8-14 | >14 |
| Echinocytosis | <2 | 2-10 | 11-50 | >50 |
| Codocytosis | <2 | 2-5 | 6-15 | >15 |
| Elliptocytosis | <1 | 1-2 | 3-8 | >8 |
| Keratocytosis | <1 | 1-2 | 3-8 | >8 |
| Acanthocytosis | <1 | 1-2 | 3-8 | >8 |
| Macrocytosis | <2 | 2-5 | 5-25 | >25 |
| Spherocytosis | <1 | 1-5 | 5-25 | >25 |
| Howell Jolly Bodies | <1 | 1-2 | 3-8 | >8 |
| Schistocytosis | <1 | 1-2 | 3-8 | >8 |
| Basophilic Stippling | <1 | 1-2 | 3-8 | >8 |
| Hypochromasia | <1 | 2-5 | 5-25 | >25 |
| Eccentrocytosis | <1 | 1-2 | 3-8 | >8 |
| Microcytosis | <1 | 2-5 | 5-25 | >25 |
| Dacrocytosis | <1 | 1-2 | 3-8 | >8 |
| Ghost Cell | <1 | 1-2 | 3-8 | >8 |
| Stomatocytosis | <1 | 1-5 | 5-25 | >25 |
| Heinz Bodies | <1 | 1-2 | 3-8 | >8 |
|  | *Quantitated as present/absent or normal density/increased density* | | | |
| Rouleaux | Present or absent | | | |
| Agglutination | Present or absent | | | |
| Siderocytosis | Present or absent | | | |
| Nucleated RBCs (nRBCs) | Normal density (<5 nRBCs/100 WBCs) or increased density (>5 nRBCs/100 WBCs) | | | |
| Atypical nuclear formations of nRBCs | Present or absent | | | |
|  | | | | |
| **Leukocyte Morphology** | **Absent or normal density (Score = 0)** | **Mildly increased (Score = 1)** | **Moderately increased (Score = 2)** | **Markedly increased (Score = 3)** |
|  | *Quantitated as percentage of leukocytes with specific morphology in ten 1000x magnified fields.* | | | |
| Neutrophil overall toxicity | <5% | 5-10% | 11-30% | >30% |
| Neutrophil cytoplasmic basophilia | <5% | 5-10% | 11-30% | >30% |
| Neutrophil Dohle bodies | <5% | 5-10% | 11-30% | >30% |
| Neutrophil foamy cytoplasm | <5% | 5-10% | 11-30% | >30% |
| Neutrophil toxic granulation | <5% | 5-10% | 11-30% | >30% |
|  | *Quantitated as present/absent or normal density/increased density* | | | |
| Reactive lymphocytes | Present or absent | | | |
| Atypical lymphocytes | Present or absent | | | |
| Granular lymphocytes | Present or absent | | | |
| Granulocyte hypersegmentation | Present or absent | | | |
| Granulocyte nuclear atypia | Present or absent | | | |
| Large unclassified cells | Present or absent | | | |
|  | | | | |
| **Platelet Morphology** | *Quantitated as present/absent or normal density/increased density* | | | |
| Macroplatelets | Present or absent | | | |
| Megakaryocytes | Present or absent | | | |
| Atypical platelet morphology | Present or absent | | | |
