# Supplementary material for: Cytomorphologic changes in blood erythrocytes, leukocytes, and platelets in dogs progressing through CHOP therapy to treat multicentric lymphoma
Source: BMC Res Notes. 2026 May 14;19:282. doi: 10.1186/s13104-026-07870-y (PMC13343946; doi:10.1186/s13104-026-07870-y)
Supplement: Supplementary file 1 — Supplementary Material 1. [file 13104_2026_7870_MOESM1_ESM.zip › Supplementary/Supplemental Table 2 Updated.docx]

**Supplemental Table 2. Erythrocyte, leukocyte, and platelet cytomorphology findings for dogs with multicentric lymphoma at diagnosis (n=20) and matched, control dogs (n=20).** Data is presented as median (range) or percentage. NV = No P value could be calculated due to complete lack of events.

| **Cytomorphology** | **Lymphoma Dogs Morphology Score** | **Control Dogs**  **Morphology Score** | ***P*** | **Lymphoma Dogs – Percentage affected** | **Control Dogs – Percentage affected** | ***P*** |
| --- | --- | --- | --- | --- | --- | --- |
| Polychromasia | 0 (0-1) | 0 (0-1) | 0.99 | 30% | 15% | 0.99 |
| Echinocytosis | 0 (0-1) | 0 (0-2) | 0.50 | 5% | 15% | 0.61 |
| Codocytosis | 0 (0-1) | 0 (0) | 0.99 | 5% | 0% | 0.99 |
| Elliptocytosis | 0 (0) | 0 (0-1) | 0.99 | 0% | 5% | 0.99 |
| Keratocytosis | 0 (0-1) | 0 (0) | 0.25 | 15% | 0% | 0.23 |
| Acanthocytosis | 0 (0) | 0 (0-1) | 0.99 | 0% | 5% | 0.99 |
| Macrocytosis | 0 (0) | 0 (0) | NV | 0% | 0% | 0.99 |
| Spherocytosis | 0 (0) | 0 (0) | NV | 0% | 0% | 0.99 |
| Howell Jolly Bodies | 0 (0-1) | 0 (0) | 0.50 | 10% | 0% | 0.49 |
| Schistocytosis | 0 (0) | 0 (0) | NV | 0% | 0% | 0.99 |
| Basophilic Stippling | 0 (0) | 0 (0) | NV | 0% | 0% | 0.99 |
| Hypochromasia | 0 (0) | 0 (0) | NV | 0% | 0% | 0.99 |
| Eccentrocytosis | 0 (0) | 0 (0) | NV | 0% | 0% | 0.99 |
| Microcytosis | 0 (0) | 0 (0) | NV | 0% | 0% | 0.99 |
| Dacrocytosis | 0 (0) | 0 (0) | NV | 0% | 0% | 0.99 |
| Ghost Cell | 0 (0) | 0 (0) | NV | 0% | 0% | 0.99 |
| Stomatocytosis | 0 (0) | 0 (0) | NV | 0% | 0% | 0.99 |
| Heinz Bodies | 0 (0) | 0 (0) | NV | 0% | 0% | 0.99 |
| Rouleaux | - | - | - | 0% | 0% | 0.99 |
| Agglutination | - | - | - | 0% | 0% | 0.99 |
| Siderocytosis | - | - | - | 0% | 0% | 0.99 |
| Elevated nucleated RBCs | - | - | - | 0% | 0% | 0.99 |
| Atypical nuclear formations of nRBCs | - | - | - | 0% | 0% | 0.99 |
| Neutrophil overall toxicity | 0 (0) | 0 (0-2) | 0.50 | 0% | 10% | 0.48 |
| Neutrophil cytoplasmic basophilia | 0 (0) | 0 (0) | NV | 0% | 0% | 0.99 |
| Neutrophil Dohle bodies | 0 (0) | 0 (0) | NV | 0% | 0% | 0.99 |
| Neutrophil foamy cytoplasm | 0 (0) | 0 (0) | NV | 0% | 0% | 0.99 |
| Neutrophil toxic granulation | 0 (0) | 0 (0-2) | 0.50 | 0% | 10% | 0.99 |
| Reactive lymphocytes | - | - | - | 20% | 0% | 0.11 |
| Atypical lymphocytes | - | - | - | 0% | 0% | 0.99 |
| Granular lymphocytes | - | - | - | 0% | 0% | 0.99 |
| Granulocyte hypersegmentation | - | - | - | 0% | 0% | 0.99 |
| Granulocyte nuclear atypia | - | - | - | 0% | 0% | NV |
| Large unclassified cells |  |  |  | 0% | 0% | NV |
| Macroplatelets | - | - | - | 0% | 0% | NV |
| Megakaryocytes | - | - | - | 0% | 0% | NV |
| Atypical platelet morphology | - | - | - | 0% | 0% | NV |
